# Molecular characterization of serous ovarian carcinoma using a multigene next generation sequencing cancer panel approach

**DOI:** 10.1186/1756-0500-7-805

**Published:** 2014-11-17

**Authors:** Nurul-Syakima Ab Mutalib, Saiful Effendi Syafruddin, Reena Rahayu Md Zain, Ahmad Zailani Hatta Mohd Dali, Ryia Illani Mohd Yunos, Sazuita Saidin, Rahman Jamal, Norfilza M Mokhtar

**Affiliations:** UKM Medical Molecular Biology Institute (UMBI), Universiti Kebangsaan Malaysia, Jalan Yaacob Latif, 56000 Cheras, Kuala Lumpur, Malaysia; Department of Pathology, Faculty of Medicine, Universiti Kebangsaan Malaysia, Jalan Yaacob Latif, 56000 Cheras, Kuala Lumpur, Malaysia; Department of Obstetrics & Gynecology, Faculty of Medicine, Universiti Kebangsaan Malaysia, Jalan Yaacob Latif, 56000 Cheras, Kuala Lumpur, Malaysia; Department of Physiology, Faculty of Medicine, Universiti Kebangsaan Malaysia, Jalan Raja Muda Abd Aziz, 50300 Kuala Lumpur, Malaysia

**Keywords:** Serous ovarian cancer, Next generation sequencing, Personal Genome Machine (PGM™), Ion AmpliSeq Cancer Hotspot Panel

## Abstract

**Background:**

High grade serous ovarian cancer is one of the poorly characterized malignancies. This study aimed to elucidate the mutational events in Malaysian patients with high grade serous ovarian cancer by performing targeted sequencing on 50 cancer hotspot genes.

**Results:**

Nine high grade serous ovarian carcinoma samples and ten normal ovarian tissues were obtained from Universiti Kebangsaan Malaysia Medical Center (UKMMC) and the Kajang Hospital. The Ion AmpliSeq™ Cancer Hotspot Panel v2 targeting “mutation-hotspot region” in 50 most common cancer-associated genes was utilized. A total of 20 variants were identified in 12 genes. Eleven (55%) were silent alterations and nine (45%) were missense mutations. Six of the nine missense mutations were predicted to be deleterious while the other three have low or neutral protein impact. Eight genes were altered in both the tumor and normal groups (*APC*, *EGFR*, *FGFR3*, *KDR*, *MET*, *PDGFRA*, *RET* and *SMO*) while four genes (*TP53*, *PIK3CA, STK11* and *KIT)* were exclusively altered in the tumor group. *TP53* alterations were present in all the tumors but not in the normal group. Six deleterious mutations in *TP53* (p.R175H, p.H193R, p.Y220C, p.Y163C, p.R282G and p.Y234H) were identified in eight serous ovarian carcinoma samples and none in the normal group.

**Conclusion:**

*TP53* remains as the most frequently altered gene in high grade serous ovarian cancer and Ion Torrent Personal Genome Machine (PGM) in combination with Ion Ampliseq™ Cancer Hotspot Panel v2 were proven to be instrumental in identifying a wide range of genetic alterations simultaneously from a minute amount of DNA. However, larger series of validation targeting more genes are necessary in order to shed a light on the molecular events underlying pathogenesis of this cancer.

**Electronic supplementary material:**

The online version of this article (doi:10.1186/1756-0500-7-805) contains supplementary material, which is available to authorized users.

## Background

Detecting cancer at an early stage or tackling it at the late stage in an efficient way is always a challenge in clinical care. Molecular approaches are now used widely at many stages of cancer management and continue to expand with the increase in the understanding of cancer biology and the correlation between genotype and phenotype. Cancer treatment has been revolutionised by the demand in molecular diagnostics to detect standard-of-care mutations with druggable targets and to predict drug response and survival [1]. The next generation sequencing (NGS) approach has become a powerful platform to complement clinical diagnosis and assist in therapeutic decision-making in cancer due to its improved sensitivity in mutation detection and fast-turnaround time compared to current gold standard methods. It has also the ability to simultaneously sequence multiple cancer-driving genes in a single assay [1].

The Sanger sequencing method, introduced in 1970, has been the gold standard for mutation analysis in cancer diagnostics [2]. However, it has a relatively low sensitivity, lower throughput with higher turnaround time and overall cost [3]. The emergence of NGS, three decades later, has overcome these drawbacks via its ability to massively sequence millions of DNA segments in parallel hence lowering the cost of sequencing per base and achieve a faster turnaround time and superior sensitivity in mutation detection [3]. As of now, whole genome sequencing is still too expensive to be used for routine diagnostics hence the option to perform targeted sequencing of exon coding regions or a subset of genes of interest offered by NGS is an attractive approach [4]. In comparison with the single gene analysis which is commonly done with the Sanger approach, the application of NGS in cancer diagnostics allows the analysis of multiple genes to identify druggable mutations and also to generate a more complete genotype of the cancer.

This study focused on ovarian cancer which is the fourth most frequently diagnosed cancer among Malaysian women, with 656 cases reported in 2007 [5]. Globally, this devastating disease is the third most common gynecological malignancy with around 225,000 new cases diagnosed in 2008 and 46.7% of them contributed by the Asian population [6]. Ovarian cancer consists of a heterogeneous group of tumors with distinct histological features, molecular characteristics and clinical behavior [7, 8]. The most common subtype of epithelial ovarian cancers is the serous carcinoma [9]. Malpica and colleagues described a 2-tier system for grading serous ovarian carcinoma i.e. as a high grade (formerly grade 2 and 3 tumors) or low grade (formerly grade 1), based mainly on the degree of nuclear atypia and mitotic rate [10]. This grading system has resulted in an effective classification of serous ovarian carcinoma as it has been proven that the low and high grade serous ovarian cancers are not only histologically different, but also exhibit distinct molecular, epidemiologic and clinical features [11].

Low grade serous ovarian carcinoma patients are younger than those with high grade counterpart, with age range from 45–57 year old and 55–65 year old, respectively [10]. In a study that compared consequence between both types of serous ovarian carcinomas using the 2-tier system, patients with low grade tumors have better survival and mortality due to disease was more rapid with high grade tumors [10]. With high grade tumors, the median survival was 1.7 years compared to 4.2 years for patients with low grade tumors [10].

From genetic perspectives, the low grade serous ovarian carcinoma is characterized by mutations in *KRAS*, *BRAF* or *ERBB2* genes; whereby approximately 66% of cases have mutations in at least one of these genes with the *ERBB2* being the least frequently mutated [12–14]. Mutual exclusivity is observed in these three genes; a tumor with a *KRAS* mutation will not have a mutation of the other two genes, and *vice versa* [9]. Comparatively, less is known about the pathogenesis of high grade serous carcinoma. Mutations of *KRAS*, *BRAF*, or *ERBB2* are infrequently detected in high grade carcinoma [12–14]. On the contrary, 80% of high grade tumors harbor *TP53* mutation and various DNA copy number aberrations [15–17]. In addition, a specific *KRAS* mutation found in low grade serous ovarian cancer has been shown to be associated with shorter survival, further adding a clinical value of NGS in cancer research [18]. Thus it is pertinent to improve our understanding of the high grade serous ovarian cancer in order to predict the prognosis of patients or for making therapeutic decisions. This study was undertaken to characterize the gene alterations in high grade serous ovarian cancer in Malaysian patients by performing targeted sequencing on 50 cancer genes with established biological functions in cancer.

## Methods

### Clinical specimens

Nine high grade serous ovarian carcinoma samples were obtained from newly diagnosed patients undergoing total abdominal hysterectomy with bilateral salphingo-oophorectomy (TAHBSO) at the Universiti Kebangsaan Malaysia Medical Center (UKMMC) and the Kajang Hospital. Ten normal ovarian tissues were also obtained from patients undergoing TAHBSO for benign gynecological diseases. The study was approved by the UKM Medical Research Ethics Committee and written informed consent was taken from the participants. None of patients had received chemotherapy or radiotherapy. The tissues were kept frozen in liquid nitrogen until subjected to cryosectioning. Hematoxylin & Eosin staining (H&E) was performed and the slides were reviewed by the pathologist. Only tissue sections that contained more than 80% tumor cell nuclei with less than 20% necrosis were included in this study. The normal specimens were confirmed to be free from tumor or inflammatory cells.

### DNA extraction and quality assessments

DNA was extracted from the tissues using the DNAeasy Blood and Tissue Kit (Qiagen, Valencia, CA). Nucleic acid quality and quantity were assessed using the Qubit Fluorometer (Invitrogen, Carlsbad, CA, USA), NanoDrop 2000 Spectrophotometer (NanoDrop Technologies, Wilmington, DE) and agarose gel electrophoresis. The highly intact and non-degraded RNA-free genomic DNA was subjected to library preparation prior to sequencing. We used 10 ng of DNA of each sample for the Ion Ampliseq library preparation.

### Library preparation

We used the Ion AmpliSeq™ Cancer Hotspot Panel v2 (Life Technologies, Guilford, CT) which allows the characterization of mutational hotspots in 50 cancer-related genes (Additional file 1: Table S1). Library preparation was performed using the Ion Ampliseq Library Kits 2.0 protocol. DNA amplification was carried out using the Ion AmpliSeq Cancer Hotspot Panel v2 and the 5x Ion AmpliseqHiFi Master Mix. Sequencing adaptors with short stretches of index sequences (barcodes) that enable sample multiplexing were ligated to the amplicons using the Ion Express Barcode Adaptors Kit (Life Technologies, Guilford, CT). The adapters-ligated amplicons (library) were purified using the Agencourt AMPure XP reagent (BD Biosciences, USA). The library was subjected to the second round of amplification using the Platinum PCR SuperMix High Fidelity and Library Amplification Primer Mix. The amplified library underwent three rounds of purification using the Agencourt AMPure XP reagent. The library was then quantified using the Bioanalyzer High Sensitivity DNA chip (Agilent Technologies Inc, Santa Clara, CA). The desired concentration for template preparation on the One Touch instrument was between 15 to 20 pM.

### Emulsion PCR and ion torrent PGM™ sequencing

The clonal amplification of the barcoded DNA library onto the ion spheres (ISPs) was carried out using emulsion PCR and the subsequent isolation of ISPs with DNA was performed using Ion OneTouch 200 Template Kit v2 DL and Ion OneTouch ES (Life Technologies, Guilford, CT) as described by the manufacturer. The polyclonal percentage and quality of the enriched, template-positive ISPs was determined using the Ion Sphere Quality Control Kit (Life Technologies, Guilford, CT). Samples with polyclonal percentage of less than 30% and enriched, template-positive ISPs of more than >80% were subjected for sequencing on the Ion Torrent Personal Genome Machine (PGM™). Enriched ISPs were subjected to sequencing on a 314 v2 Ion Chip (two samples per chip) using Ion PGM™ Sequencing 200 kit v2 (Life Technologies, Guilford, CT). A cut-off with a quality score of Q17 (a quality score of 2% errors, corresponding to 1 base error allowed per 50 bases) was used as a measure of successful sequencing.

### Validation using sanger sequencing

Variants identified were validated using the Sanger sequencing method. Primers corresponding to the alteration sites were purchased from Life Technologies. The primer pairs chosen are HS00424883_CE, HS00432201_CE and HS00346578_CE. Briefly, PCR products were generated and cycle sequencing was performed using the Big Dye Terminator V3.1 reagent (Life Technologies, Guilford, CT). The cycle sequencing products were then processed using ethanol precipitation and sequencing was carried out using the ABI 3130xl capillary electrophoresis (Life Technologies, Guilford, CT). The results were analyzed using the Basic Local Alignment System Tool (BLAST).

### Bioinformatics analysis

#### Read mapping and variants calling

Data from sequencing runs from Ion Torrent PGM™ were automatically transferred to the Torrent Server hosting the Torrent Suite Software that processed the raw voltage semiconductor sequencing data into DNA base calls. The Torrent Suite Software utilizes the Torrent Browser that includes TMAP alignment and Torrent Variant Caller for alignment and variant detection. Data were aligned against Human hg19 database. The Ion Reporter Software (Life Technologies, Guilford, CT) was used to perform variant calling and mapping.

#### Filtering of the variants called

A number of steps were used to filter nucleotide variants identified in the screening; (a) variants that are not annotated as pathogenic or probable pathogenic variants were excluded, (b) variants called in both normal and serous ovarian cancer genome were excluded and (c) variants representing probable mapping ambiguities were excluded. Manual and thorough observation of the variants using the Integrated Genomic Viewer (IGV) was performed to exclude false variants [19].

### Predicting the functional significance of nonsynonymous mutations

Nonsynonymous missense mutations called were evaluated by *in silico* analysis using the TransFIC (TRANSformed Functional Impact for Cancer (TransFIC) method (http://bg.upf.edu/transfic/). The method transforms Functional Impact scores taking into account the differences in basal tolerance to germline structural number variations of genes that belongs to the different functional classes. This transformation allows the use of the scores provided by well-known tools (SIFT, Polyphen2 and Mutation Assessor) to rank the functional impact of cancer somatic mutations. Mutations with a greater TransFIC values are more likely to be the cancer drivers.

### Cancer genes annotation

Annotation was performed using Oncotator (http://www.broadinstitute.org/oncotator/), a web application for annotating human genomic point mutations and indels with data relevant to cancer researchers.

### Statistical analysis

We utilized the Fisher's exact test to define significant values in a number of altered genes and total variants between tumor and normal samples using the 2 × 2 contingency tables and the GraphPad QuickCalcs Online Calculator for Scientists (http://www.graphpad.com/quickcalcs/index.cfm). All p values are two-sided and statistical significance is denoted by p <0.05.

### Integrative analysis using the ICGC data portal

We used the International Cancer Genome Consortium (ICGC) Data Portal [20], a web tool for exploring, visualizing and analyzing multi-dimensional cancer genomics data, to interactively explore genetic alterations across samples, genes and pathways in both the ICGC and our datasets.

## Results

### Epidemiological characteristics

The epidemiological features of the studied subjects are presented in Table 1. The median age was 57 years for the patients with serous ovarian cancer and 52 years for the normal controls.

**Table 1:** **Clinical information of samples**

Description	N (%)
Diagnosis (tumour group)	
Ovarian serous cyst adenocarcinoma (poorly differentiated) with anaplasia	1 (11%)
Metastatic high grade serous adenocarcinoma of the ovary	1 (11%)
Bilateral ovarian papillary serous cystadenocarcinoma, moderately differentiated	1 (11%)
Ovarian serous adenocarcinoma	6 (67%)
Age	
< 50 year-old	4 (21%)
> 50 year-old	12 (63%)
Unknown	3 (16%)
Ethnic	
Malay	14 (74%)
Chinese	4 (21%)
Indian	1 (5%)

### Technical performance of the Ion AmpliSeq™ Cancer Hotspot Panel v2

The Ion AmpliSeq™ Cancer Hotspot Panel v2 contains 207 primer pairs that cover the “mutation-hotspot region” in 50 most common cancer-associated genes (Additional file 1: Table S1). The average sample loading obtained was 83.8% (range 76% - 91%). The total reads ranged from 220,000 – 420,000 reads with an average read length of 112 bp. The details on the loading percentage, number of reads and sequenced bases for each of the samples are summarized in Additional file 1: Table S2. On average, the sequencing coverage for each of the regions is >1000×.

### Summary of identified variants

We identified a total of 20 variants in 12 genes. Eight genes were altered in both the tumor and normal groups (*APC*, *EGFR*, *FGFR3*, *KDR*, *MET*, *PDGFRA*, *RET* and *SMO*) while four genes (*TP53*, *PIK3CA, STK11* and *KIT)* showed presence of alterations in only the cancer samples. From the total of 20 variants, 11 (55%) were silent alterations and nine (45%) were missense mutations. Six of the nine missense mutations were predicted to have deleterious impact on the proteins while the other three have low or neutral protein impact. Ten variants were identified from eight genes in both the tumor and normal groups (*APC*, *EGFR*, *FGFR3*, *KDR*, *MET*, *PDGFRA*, *RET* and *SMO*). However upon annotation, these variants resulted in no amino acid changes (silent alteration), have neutral protein impact or presented in normal population according to dbSNP database version 38 (minimal allele frequency >2%).

**Figure 1:**
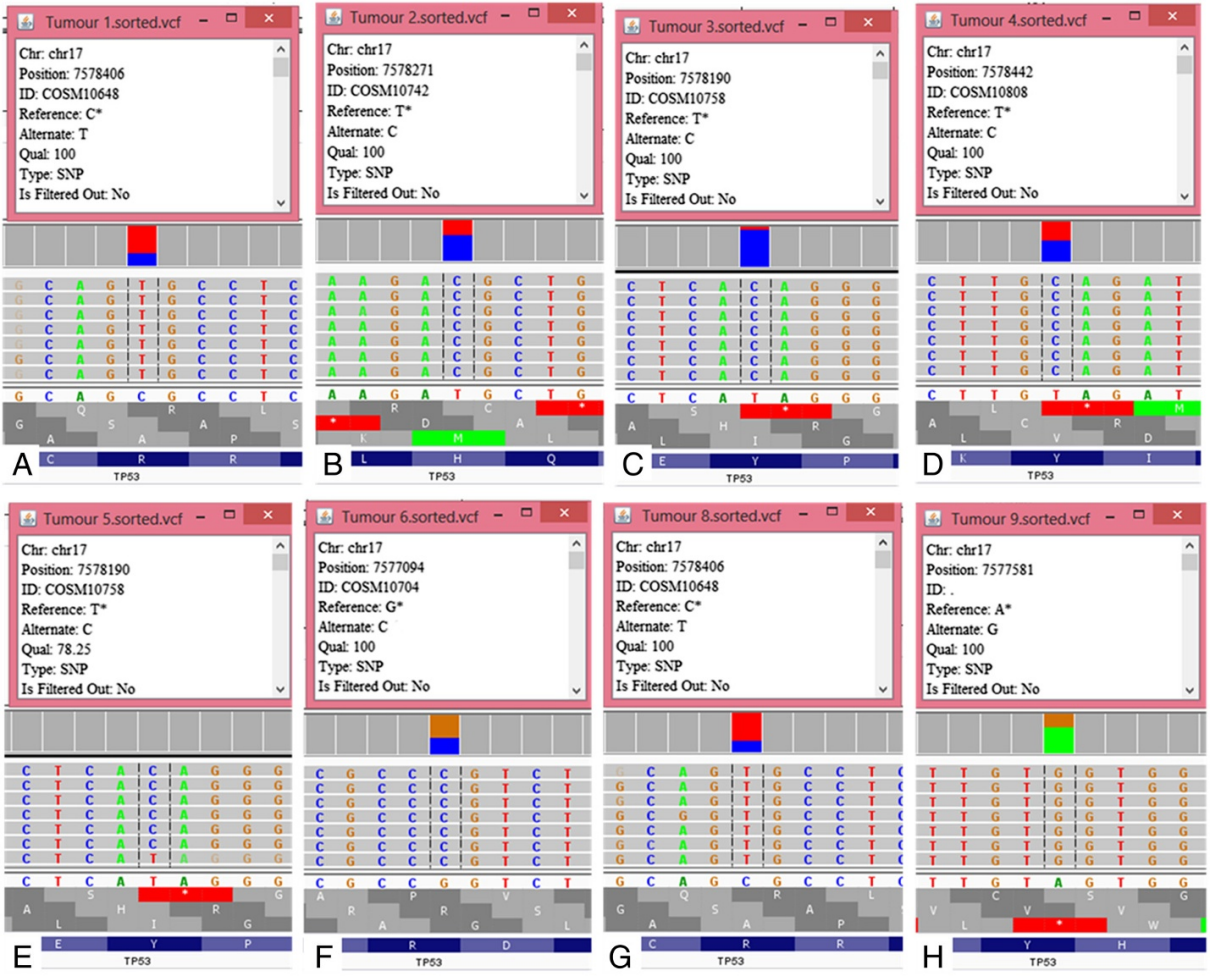
**Deleterious mutations identified by targeted next generation sequencing in serous ovarian carcinoma.** Representation of the reads aligned to the reference genome of **A)** Tumor 1; **B)** Tumor 2; **C)** Tumor 3; **D)** Tumor 4; **E)** Tumor 5; **F)** Tumor 6; **G)** Tumor 8 and **H)** Tumor 9; as provided by the Integrative Genomics Viewer V 2.3 software [19].

Base transitions (purine-purine and pyrimide-pyrimidine) were more frequent than transversions (purine-pyrimidine, *vice versa*) (Additional file 1: Figure S1). At least four genes were altered in each tumor and normal samples. The details on the variant frequency are shown in Table 2. Our results showed that *TP53* alterations were present in all the tumors but not in the normal group. We further analyzed the types of alteration identified in the high grade serous ovarian cancer. Table 3 illustrates the variant distribution of the high grade serous ovarian carcinoma in our cohort of patients. After manual filtration, eight of the nine high grade serous ovarian carcinoma samples harboured one deleterious mutation and all of the deleterious mutations were found in *TP53* as shown in Table 4. The remaining one high grade serous ovarian carcinoma sample (T7) did not have any deleterious mutation. There was no deleterious mutation found in the 10 normal samples (Additional file 1: Table S3). In addition, the cancer samples showed significantly more altered genes than the normal group (average seven altered genes in tumor group versus 4.8 altered genes in normal group; p value = 0.0001). Six deleterious mutations in *TP53* with different codon and protein changes were identified in eight serous ovarian carcinoma samples (Figure 1).

**Table 2:** **Variant frequency in high grade serous ovarian carcinoma versus normal ovary**

Gene/Variant frequency	APC	EGFR	FGFR3	KDR	KIT	MET	PDGFRA	PIK3CA	RET	SMO	STK11	TP53
Tumour	89%	67%	100%	89%	22%	11%	100%	11%	67%	22%	22%	100%
Normal	100%	30%	100%	70%	0%	10%	100%	0%	60%	10%	0%	0%
p value	0.4737	0.1789	1	0.5820	0.2105	1	1	0.4737	1	0.5820	0.2105	0.0001***

***Statistically significant value.

*TP53* alterations are statistically significantly more frequent in serous ovarian carcinoma.

**Table 3:** **Variant distribution of high grade serous ovarian carcinoma**

Sample ID/Gene	T1	T2	T3	T4	T5	T6	T7	T8	T9
**APC**	-	**112175770G > A	**112175770G > A	*112175770G > A	*112175770G > A	*112175770G > A	*112175770G > A	**112175770G > A	*112175770G > A
**EGFR**	*55249063G > A	*55249063G > A	*55249063G > A	*55249063G > A	*55249063G > A	-	-	*55249063G > A	-
**FGFR3**	**1807894G > A	**1807894G > A	**1807894G > A	**1807894G > A	**1807894G > A	**1807894G > A	**1807894G > A	**1807894G > A	**1807894G > A
**KDR**	*55972974T > A	*55972974T > A	*55972974T > A	-	**55972974T > A	**55972974T > A	*55972974T > A	*55972974T > A	*55972974T > A
**KIT**	*55593481A > G	-	-	*55593464A > C	-	-	-	-	-
**MET**	-	-	-	-	-	-	-	*116339672C > T	-
**PDGFRA**	**55141055A > G	**55141055A > G	**55141055A > G	**55141055A > G	**55141055A > G	**55141055A > G	**55141055A > G	**55152040C > T ** 55141055A > G	**55141055A > G
	*55152040C > T		*55152040C > T			*55152040C > T			
**PIK3CA**	-	-	-	-	-	*178952020C > T	-	-	-
**RET**	*43613843G > T	*43613843G > T	*43613843G > T	-	-	*43613843G > T	*43613843G > T	-	**43613843G > T
**SMO**	-	*128845088A > G	*128845088A > G	-	-	-	-	-	-
**STK11**	-	-	-	*1223125C > G	-	*1223125C > G	-	-	-
**TP53**	Deleterious* 7578406C > T	Deleterious* 7578271T > C	*7577035T > C Deleterious *7578190T > C	Deleterious* 7578442T > C	Deleterious* 7578190T > C	Deleterious* 7577094G > C	*7577035T > C	Deleterious* 7578406C > T	Deleterious* 7577581A > G
**# Altered genes**	7	8	8	7	6	8	6	7	6
**# Del variant**	1	1	1	1	1	1	0	1	1
**# Total variants**	8	8	10	7	6	9	6	8	6

**Homozygous.

*Heterozygous.

Del = Deleterious.

**Table 4:** **Deleterious mutations identified in high grade serous ovarian cancer samples**

Sample ID	Gene	Mutation	Codon change	Protein change	Type	dbSNP ID/COSMIC ID	transFIC prediction
**T1**	TP53	g.chr17:7578406C > T	c.(523–525)CGC > CAC	p.R175H	SNP	rs28934578	Driver mutation
**T2**	TP53	g.chr17:7578271T > C	c.(577–579)CAT > CGT	p.H193R	SNP	COSM10742	Driver mutation
**T3**	TP53	g.chr17:7578190T > C	c.(658–660)TAT > TGT	p.Y220C	SNP	rs121912666	Driver mutation
**T4**	TP53	g.chr17:7578442T > C	c.(487–489)TAC > TGC	p.Y163C	SNP	rs148924904	Strongly affecting mutation
**T5**	TP53	g.chr17:7578190T > C	c.(658–660)TAT > TGT	p.Y220C	SNP	rs121912666	Driver mutation
**T6**	TP53	g.chr17:7577094G > C	c.(844–846)CGG > GGG	p.R282G	SNP	rs28934574	Driver mutation
**T8**	TP53	g.chr17:7578406C > T	c.(523–525)CGC > CAC	p.R175H	SNP	rs28934578	Driver mutation
**T9**	TP53	g.chr17:7577581A > G	c.(700–702)TAC > CAC	p.Y234H	SNP	COSM11152	Driver mutation

SNP = single nucleotide polymorphism.

### Correlation with ICGC datasets

We compared the deleterious mutations obtained from this study with those in the ICGC Data Portal. Table 5 illustrates the correlations and occurrences of those deleterious mutations. Among all of the deleterious mutations found in this study, four have been reported in serous ovarian carcinoma from the consortium (*TP53* p.R175H, p.H193R, p.Y220C, p.Y163C and p.R282G) with the frequency ranging from 0.36 - 2.16% [20]. Meanwhile, the *TP53* p.R282G and p.Y234H have not been reported to be present in the ICGC’s serous ovarian carcinoma cases but have been detected in lung cancer (0.9%), breast cancer (0.11%) and renal cancer (0.25%) [20].

**Table 5:** **Comparison of** ***TP53*** **deleterious mutations in this study with ICGC data**

	p.R175H	p.H193R	p.Y220C	p.Y163C	p.R282G	p.Y234H
ICGC mutation ID	MU7870	MU13250	MU4807	MU10836	MU34029	MU622929
**Occurrences**	Affects 66 distinct donors across 12 cancer projects	Affects 9 distinct donors across 4 cancer projects	Affects 24 distinct donors across 9 cancer projects	Affects 13 distinct donors across 7 cancer projects	Affects 2 distinct donors across 2 cancer projects	Affects 1 distinct donors across 1 cancer project
**Occurrences in serous ovarian cancer (ICGC)**	6/278 (2.16%)	1/278 (0.36%)	4/278 (1.44%)	1/278 (0.36)	None reported	None reported
**Occurrences in serous ovarian cancer (this study)**	2/10 (20%)	1/10 (10%)	2/10 (20%)	1/10 (10%)	1/10 (10%)	1/10 (10%)
**Other cancers involved**	Brain cancer (5/269 (1.86%))	Endometrial cancer (6/766 (0.78%))	Brain cancer (2/269 (0.74%))	Breast cancer (2/117 (1.71%))	Lung cancer (1/111 (0.9%))	Renal cancer (1/407 (0.25%))
	Breast cancer (15/766 (1.96%))	Lung cancer (1/246 (0.41%))	Breast cancer (2/117 (1.71%))	Breast cancer (2/766 (0.26%))	Breast cancer (1/943 (0.11%))	
	Colon cancer 15/261 (5.75%))	Breast cancer (1/178 (0.56%))	Breast cancer (6/766 (0.78%))	Colon cancer (1/261 (0.38%))		
	Endometrial cancer (2/246 (0.81%))		Colon cancer 2/261 (0.77%))	Liver cancer (3/213 (1.41%))		
	Esophageal cancer (2/22 (9.09%))		Endometrial cancer (3/246 (1.22%))	Lung cancer (3/178 (1.69%))		
	Gastric cancer (1/10 (10.00%))		Liver cancer (1/42 (2.38%))	Pancreatic cancer (1/187 (0.53%))		
	Oral cancer (2/50 (4.00%))		Lung cancer (2/178 (1.12%))			
	Pancreatic cancer (8/187 (4.28%) and 3/85 (3.53%))		Pancreatic cancer (2/187 (1.07%))			
	Pediatric brain tumors (1/193 (0.52%))					
	Rectal cancer (6/109 (5.50%))					

Frequency of *TP53* mutations from this study in comparison with ICGC data. Information was extracted from the data portal (Zhang *et al*. [20]) and no post-analysis modification was made.

## Discussion

In this study, we analyzed the base alteration status of 50 cancer-related genes using a targeted NGS approach on nine high grade serous ovarian carcinoma and 10 normal ovaries. Since all cancers included in our study were of high grade serous histology, we have avoided any false correlation that might have resulted from a study of mixed histological types. We observed that transitions and substitutions were more frequent than transversions and this is in agreement with other studies on the patterns of somatic mutation in human cancer genomes [21, 22].

We found that *TP53* alterations were the most common alterations identified in our local patients with high grade serous ovarian carcinoma. In addition, the majority of alterations identified in *TP53* were predicted to be deleterious and four of the mutations were the hotspot mutations (p.R175H, p.H193R, p.Y220C and p.Y163C). These deleterious mutations in *TP53* were detected in eight of the nine cancer samples. Our findings were in agreement with other studies on high grade serous ovarian carcinoma in which more than 80% of cases harboured *TP53* mutations [12, 15, 17]. We could conclude that *TP53* mutations remain the most consistent genomic feature in high grade serous ovarian carcinoma.

*TP53* which encodes the tumor suppressor protein p53, is among the most frequently mutated genes in human cancers [23]. The ubiquitous presence of *TP53* mutations in ovarian cancer has been suggested more than 20 years ago, particularly in those with serous histology [24]. Whilst some tumor suppressor genes, such as *APC* or *BRCA1*, are frequently deactivated by the frame shift or nonsense mutations, the missense mutation is the predominant type of mutation in *TP53* in human tumors [25]. These mutations are identified mainly in exons 4–9, which encode the DNA-binding domain of the protein [26]. We identified seven mutations in *TP53* DNA binding domain from which six were missense mutations (86%) and one silent alteration (14%). These observations confirmed previous findings that missense mutation was the predominant alteration in *TP53* in tumors [25].

A tumor cell with a *TP53* missense mutation could result in full-length p53 proteins which have prolonged half-life and tend to accumulate in the tumor cells [25]. These mutant proteins were hypothesized to possess the ability to influence tumor progression. Oren and Rotter revealed that mutant p53 proteins can bind and deactivate other related proteins such as p63 and p73 [27]. This tumorigenic activity of mutant p53 has been described as gain-of-function (GOF), which was shown to coerce tumor cells toward migration, invasion and metastasis in mouse models as demonstrated by several other studies [28, 29]. A study by Kang and colleagues also demonstrated that high grade serous ovarian cancer patients with GOF mutant p53 frequently showed resistance against platinum-based treatment and were prone to distant metastasis [25].

We also observed low impact alteration in *KIT* and *STK11* exclusively in the tumor group which resulted from base transversions. The *KIT* p.K546K, a silent alteration resulted from A > G transversion at position 55593481, was identified in sample T1 and was not detected in any ICGC studies [20]. Another alteration in *KIT,* the p.M541L, is a missense mutation resulting from A > C transversion at position 55593464 found in sample T4, was also detected in a patient with gastric cancer patient as reported in the ICGC’s portal [20]. The *STK11* p.F354L, a missense mutation identified in sample T4 and T6, resulted from a C > G transversion at position 1223125. To our knowledge, this alteration has not been detected in any of the published data from the consortium studies. The significance of these low impact alterations is still yet to be identified. However, the effort towards the development of personalized medicine must be guided by a comprehensive look at the mutational landscape of each tumor, hence these low impact alterations should not be disregarded.

The small sample size is an obvious limitation of this study and this could affect our interpretation of the results. Therefore a larger series of validation including bigger sample size, different histological subtypes, inclusion of germline DNA derived from the same sample are indispensable in order to fully understand the genetic events underlying this cancer. In addition, this panel is targeting “hotspot” region of genes that are frequently mutated in human cancer thus other infrequently altered genes but of significance to serous ovarian cancers might have been excluded such as *ARID1A*, *BRCA1*, *BRCA2*, *CSMD3*, *NF1*, *CDK12*, *FAT3* and *GABRA6* [30]. Furthermore, variation in cancers may not be reflected in the sequence variation alone. Other important factors such as gene expression (coding and non-coding), DNA methylation as well as influence of genomic rearrangements such as copy number and structural variations are unable to be reliably addressed using this approach.

## Conclusions

Our findings revealed a relatively small number of somatic alterations in high grade serous ovarian cancer using this commercially available cancer hotspot panel. An extensive somatic mutation screening targeting more genes using the Ion Ampliseq™ Comprehensive Cancer Panel (CCP) or whole exome sequencing might be a preferred approach for future research, particularly in this disease. Undoubtedly, the implementation of this hotspot panel in routine molecular diagnostics for high grade serous ovarian carcinoma definitely requires validation in larger series of samples. Nevertheless, its superior performance in identifying a wide range of genetic alterations simultaneously from a minute amount of DNA can facilitate the evaluation of tumor-specific treatment susceptibility and individual prognosis.

## Electronic supplementary material

Additional file 1: Table S1: List of genes covered in the Cancer Hotspot Panel v2. **Table S2.** Sequencing outputs from Ion Torrent PGM. **Table S3.** Variant distribution of normal ovary. **Figure S1.** Category of DNA substitution. Majority of substitution were of transition substitution (purine-purine and pyrimidine-pyrimidine). (DOCX 200 KB)
