# Supplementary material for: Molecular characterization of serous ovarian carcinoma using a multigene next generation sequencing cancer panel approach
Source: BMC Res Notes. 2014 Nov 17;7:805. doi: 10.1186/1756-0500-7-805 (PMC4242548; doi:10.1186/1756-0500-7-805)
Supplement: Supplementary file 1 — Additional file 1: Table S1: List of genes covered in the Cancer Hotspot Panel v2. Table S2. Sequencing outputs from Ion Torrent PGM. Table S3. Variant distribution of normal ovary. Figure S1. Category of DNA substitution. Majority of substitution were of transition substitution (purine-purine and pyrimidine-pyrimidine). (DOCX 200 KB) [file 13104_2014_3327_MOESM1_ESM.docx]

Supplementary Table 1. List of genes covered in the Cancer Hotspot Panel v2.

| *ABL1* | *AKT1* | *ALK* | *APC* | *ATM* |
| --- | --- | --- | --- | --- |
| *BRAF* | *CDH1* | *CDKN2A* | *CSF1R* | *CTNNB1* |
| *EGFR* | *ERBB2* | *ERBB4* | *EZH2* | *FBXW7* |
| *FGFR1* | *FGFR2* | *FGFR3* | *FLT3* | *GNA11* |
| *GNAS* | *GNASQ* | *HNF1A* | *HRAS* | *IDH1* |
| *JAK2* | *JAK3* | *IDH2* | *KDR* | *KIT* |
| *KRAS* | *MET* | *MLH1* | *MPL* | *NOTCH1* |
| *NPM1* | *NRAS* | *PDGFRA* | *PIK3CA* | *PTEN* |
| *PTPN11* | *RB1* | *RET* | *SMAD4* | *SMARCB1* |
| *SMO* | *SRC* | *STK11* | *TP53* | *VHL* |

Supplementary Table 2. Sequencing outputs from Ion Torrent PGM.

| **Samples** | **ISPs Loading** | **Total Number of Reads** | **Total bases (>Q20)** | **Mean Read Length** |
| --- | --- | --- | --- | --- |
| T1 | 89% | 335 815 | 34.7 Mbp | 110 |
| T2 | 76% | 228 245 | 23.3 Mbp | 109 |
| T3 | 84% | 366 964 | 39.4 Mbp | 113 |
| T4 | 79% | 325 935 | 34.5 Mbp | 113 |
| T5 | 76% | 269 410 | 28.6 Mbp | 112 |
| T6 | 91% | 404 366 | 42.9 Mbp | 113 |
| T7 | 87% | 310 015 | 33.2 Mbp | 115 |
| T8 | 85% | 327 051 | 34.5 Mbp | 114 |
| T9 | 85% | 282 500 | 28.6 Mbp | 110 |
| N1 | 89% | 346 322 | 36.6 Mbp | 112 |
| N2 | 91% | 318 912 | 34.2 Mbp | 114 |
| N3 | 85% | 259 365 | 27.2 Mbp | 112 |
| N4 | 76% | 311 194 | 32.1 Mbp | 110 |
| N5 | 84% | 374 656 | 40.8 Mbp | 115 |
| N6 | 79% | 262 802 | 27.9 Mbp | 113 |
| N7 | 86% | 221 267 | 22.8 Mbp | 112 |
| N8 | 76% | 299 495 | 32.3 Mbp | 114 |
| N9 | 87% | 418 675 | 44.3 Mbp | 113 |
| N10 | 85% | 358 632 | 37.1 Mbp | 114 |

Mbp = million base pair

ISP = Ion Sphere™ particles

Q20 = an error rate of 1 in 100, with a corresponding call accuracy of 99%.

Supplementary Table 3. Variant distribution of normal ovary.

| **Sample ID / Gene** | **N1** | **N2** | **N3** | **N4** | **N5** | **N6** | **N7** | **N8** | **N9** | **N10** |
| --- | --- | --- | --- | --- | --- | --- | --- | --- | --- | --- |
| **APC** | * 112175770G>A | ** 112175770G>A | * 112175770G>A | ** 112175770G>A | * 112175770G>A | ** 112175770G>A | ** 112175770G>A | ** 112175770G>A | ** 112175770G>A | ** 112175770G>A |
| **EGFR** | - | - | - | - | - | - | * 55249063G>A | * 55249063G>A | *  55249063G>A | - |
| **FGFR3** | ** 1807894G>A | ** 1807894G>A | ** 1807894G>A | ** 1807894G>A | ** 1807894G>A | ** 1807894G>A | ** 1807894G>A | ** 1807894G>A | **  1807894G>A | **  1807894G>A |
| **KDR** | ** 55972974T>A |  | * 55972974T>A | * 55972974T>A | * 55972974T>A | * 55972974T>A |  |  | **  55972974T>A | **  55972974T>A |
| **MET** | - | - | - | - | * 116340262A>G  *  116339672C>T | - | - | - | - | - |
| **PDGFRA** | ** 55141055A>G | ** 55141055A>G  * 55152040C>T | ** 55141055A>G  * 55152040C>T | ** 55141055A>G | ** 55141055A>G | ** 55141055A>G  * 55152040C>T | ** 55141055A>G  * 55152040C>T | ** 55141055A>G  * 55152040C>T | **  55141055A>G  *  55152040C>T | **  55141055A>G |
| **RET** | * 43613843G>T | * 43613843G>T | - | - | - | - | - | - | - |  |
| **SMO** |  |  |  |  |  |  |  |  |  | **  128845088A>G |
| **# Altered genes** | 5 | 4 | 4 | 4 | 5 | 4 | 4 | 4 | 5 | 5 |
| **# Del variants** | None | | | | | | | | | |
| **# Total variants** | 5 | 5 | 5 | 4 | 6 | 5 | 5 | 5 | 6 | 5 |

**Homozygous

*Heterozygous

Del = Deleterious


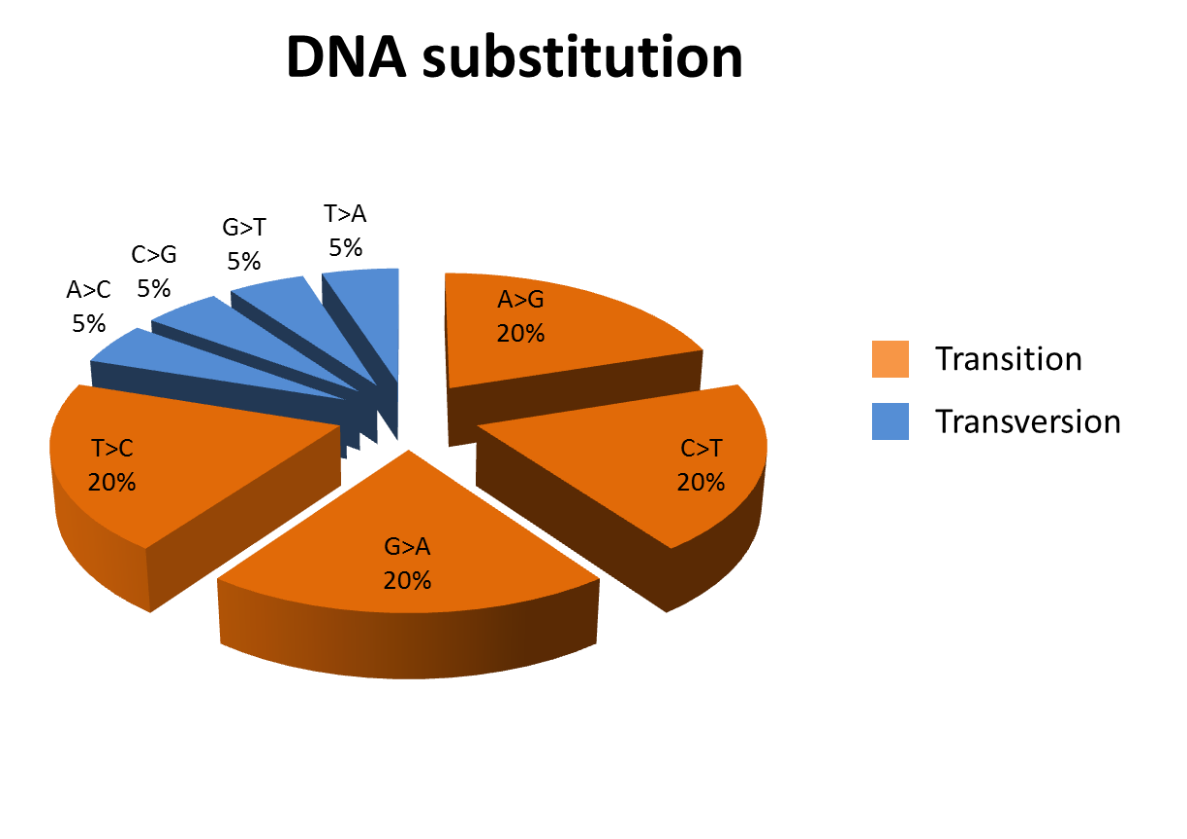


**Supplementary Figure 1.** Category of DNA substitution. Majority of substitution were of transition substitution (purine-purine and pyrimidine-pyrimidine).
